# Geriatric assessment in patients aged 70 and over considered for CAR-T therapy: Adescriptive study

**DOI:** 10.1093/geroni/igaf122.3203

**Published:** 2025-12-31

**Authors:** Rachel Boisvert

**Affiliations:** Université Laval, Quebec, Quebec, Canada

## Abstract

Geriatric oncology addresses the challenges of treating elderly cancer patients, who are often undertreated due to complex care needs. CAR-T therapy, effective for some hematologic cancers, presents significant side effects that complicate its use in patients over 70. Comprehensive geriatric assessment may help guide treatment decisions in this population. This study aims to describe the profile of elderly patients referred for potential CAR-T therapy, assess the impact of the geriatric evaluation on treatment decisions, examine the occurrence of ICANS and cytokine release syndrome in relation to frailty, and monitor clinical outcomes such as hospitalizations and mortality. This retrospective study will include patients aged 70 and over referred to the Quebec Geriatric Clinic for pre-treatment assessment before May 2025. Data will be collected from medical records, including patient profiles, initial treatment plans, recommendations from geriatric oncology, final therapeutic plans, and clinical outcomes. No complex statistical analysis will be performed due to the limited sample size (approximately 30 patients). Results will be presented descriptively. We expect that the geriatric evaluation will lead to modifications in treatment plans and identify risk factors for severe adverse effects, helping to refine eligibility criteria for CAR-T therapy in elderly patients. The findings could enhance clinical practices by promoting personalized care in geriatric oncology. This study seeks to demonstrate the importance of integrating a complete geriatric evaluation into treatment planning for elderly cancer patients, optimizing care and decision-making for CAR-T therapy.

